# Multiple comparisons of precipitation variations in different areas using simultaneous confidence intervals for all possible ratios of variances of several zero-inflated lognormal models

**DOI:** 10.7717/peerj.12659

**Published:** 2021-12-20

**Authors:** Patcharee Maneerat, Sa-Aat Niwitpong

**Affiliations:** 1 Department of Applied Mathematics, Rajabhat Uttaradit University, Uttaradit, Thailand; 2 Department of Applied Statistics, King Mongkut’s University of Technology North Bangk, Bangkok, Thailand

**Keywords:** Precipitation variation, Ratio of variances, Bayesian approach, Parametric bootstrap approach, Simulation, Rainfall data, Beta prior

## Abstract

Flash flooding and landslides regularly cause injury, death, and homelessness in Thailand. An advancedwarning system is necessary for predicting natural disasters, and analyzing the variability of daily precipitation might be usable in this regard. Moreover, analyzing the differences in precipitation data among multiple weather stations could be used to predict variations in meteorological conditions throughout the country. Since precipitation data in Thailand follow a zero-inflated lognormal (ZILN) distribution, multiple comparisons of precipitation variation in different areas can be addressed by using simultaneous confidence intervals (SCIs) for all possible pairwise ratios of variances of several ZILN models. Herein, we formulate SCIs using Bayesian, generalized pivotal quantity (GPQ), and parametric bootstrap (PB) approaches. The results of a simulation study provide insight into the performances of the SCIs. Those based on PB and the Bayesian approach via probability matching with the beta prior performed well in situations with a large amount of zero-inflated data with a large variance. Besides, the Bayesian based on the reference-beta prior and GPQ SCIs can be considered as alternative approaches for small-to-large and medium-to-large sample sizes from large population, respectively. These approaches were applied to estimate the precipitation variability among weather stations in lower southern Thailand to illustrate their efficacies.

## Introduction and Motivation

In early 2021, approximately 186,300 people in lower southern Thailand were affected by heavy rainfall resulting in flash flooding, landslides, and windstorms, as reported by Thailand’s Department of Disaster Prevention and Migration (DDPM) (Thailand, 2021). Four provinces in the lower southern region of Thailand were affected by flooding: Songkhla (60 households), Pattani (2,810 households), Yala (12,082 households), and Narathiwat (22,308 households). Meanwhile, landslides occurred in Yala and Narathiwat that affected approximately 57 households (Thailand, 2021). Unfortunately, these natural disasters resulted in deaths and injuries (David, 2021).

It would be possible to reduce the impact of natural disasters if governmental organizations had an early warning system that could be triggered to warn people in high-risk areas in advance of impending catastrophes. Rainfall dispersion data can provide essential information indicating imminent flooding when variation is high by analyzing historical precipitation data. Importantly, it could also be used to predict precipitation variation in each area. From the historical evidence of flooding in lower southern Thailand, the precipitation data in four areas are inflated with zero observations, while the non-zero precipitation records are log-normally distributed, as can be seen in An Empirical Application Section. These properties indicate that precipitation data obey the assumptions for a zero-inflated lognormal (ZILN) distribution and can be modeled accordingly.

The ZILN model, also referred to as the delta-lognormal model, is appropriate for modeling right-skewed data with a proportion of zero (Aitchison & Brown, 1963; Fletcher, 2008; Wu & Hsieh, 2014; Hasan & Krishnamoorthy, 2018; Maneerat, Niwitpong & Niwitpong, 2019). Variance is a dispersion measure of probability used in statistical inference for both point and interval (*e.g.*, confidence interval: CI) estimation. Several researchers have formulated point and interval estimates *via* various approaches. For example, Burdick & Graybill (1984) established CIs for linear combinations of the variance components using the unbalanced one-way classification model and the Graybill-Wang procedure by considering the inequality of the design (Graybill & Wang, 1980). Ciach & Krajewski (1999) estimated the radar-raingauge difference variances which can be separated into the area-point ground raingauge originating from resolution difference between them, and the error of the radar area-average rainfall estimate. Another important approach for variance estimation is bootstrapping based on t-statistics to formulate nonparametric CIs for a single variance and the difference between variances, which was used to estimate the variance in insurance data for properties (Cojbasic & Tomovic, 2007). Bebu & Mathew (2008) used a modified single log-likelihood ratio procedure to construct CIs for the ratio of bivariate lognormal variances and applied it to compare variation in health care costs. Cojbasic & Loncar (2011) suggested Hall’s bootstrapped-t method for constructing one-sided CIs (lower and upper endpoint CIs) for the variances of skewed distributions and illustrated the efficacy of their method by analyzing revenue variability within the food retail industry.

Later, Herbert et al. (2011) suggested an analytical method for the difference between two independent variances that performed well even with small unequal sample sizes and highly skewed leptokurtic data; they used data from a randomized trial for a cholesterol-lowering drug to portray the efficacies of their proposed methods. Harvey & Merwe (2012) revealed that a Bayesian CI based on the highest posterior density outperformed one based on the equal-tailed interval for the variance of lognormal distribution with zero observations. Maneerat, Niwitpong & Niwitpong (2020) showed that the highest posterior density interval based on a probability matching prior produced the narrowest interval with correct coverage for comparing delta-lognormal variances; they applied it to estimate the difference between rainfall variability in the lower and upper northern regions of Thailand. Recently, Bayesian credible intervals based on a non-informative prior were presented by Maneerat, Niwitpong & Niwitpong (2021a) for the single variance of a delta-lognormal model that was used on daily rainfall records.

Nevertheless, no studies have yet been conducted on simultaneous CIs (SCIs) for pairwise comparisons of the variances of several ZILN models, and so we addressed our research toward filling this gap. Hence, we estimated all possible ratios of variances of several ZILN models by using SCIs based on Bayesian, parametric bootstrap (PB), and generalized pivotal quantity (GPQ) approaches. The reasons for choosing them are that the Bayesian and PB approaches can be used to construct CIs capable of handling situations with large differences in the variances and high proportion of zero values of delta-lognormal models, respectively (Maneerat, Niwitpong & Niwitpong, 2020), while CI based on the GPQ approach perform quite well when the variance was large maneeratEstimatingFishDispersal2020. Their efficacies were determined *via* simulation studies and precipitation data from four areas of the lower southern region of Thailand in terms of the coverage rate (CR), the lower error rate (LER), the upper error rate (UER), and the average width (AW).

## Model and methods

### Model

For *h* groups, *d*_*i*_; *i* = 1, 2, …, *h*, denotes the probability of having zero observations while the remaining probability for non-zero observations, ${d}_{i}^{{^{\prime}}}=1-{d}_{i}$, follows a lognormal distribution denoted as $\text{LN}({\mu }_{i},{\sigma }_{i}^{2})$ with mean *μ*_*i*_ and variance ${\sigma }_{i}^{2}$. For random samples from the groups, let *Y*_*i*_ = (*Y*_*i*1_, *Y*_*i*2_, …., *Y*_*in*_*i*__) denote a ZILN variate based on *n*_*i*_ observations from group *i* with the probability density function given by

(1) \begin{eqnarray*}g({y}_{i};{d}^{{^{\prime}}},{\mu }_{i},{\sigma }_{i}^{2})={d}_{i}+{d}_{i}^{{^{\prime}}}{y}^{-1}(2\pi {\sigma }_{i}^{2})^{-1/2}\exp \nolimits \left\{ - \frac{(\ln \nolimits {y}_{i}-{\mu }_{i})^{2}}{2{\sigma }_{i}^{2}} \right\} .\end{eqnarray*}

For *Y*_*i*_ = 0, the number of zero observations *n*_*i*0_ follows a binomial distribution with sample size *n*_*i*_ and the probability of having zero observations *d*_*i*_, where *n*_*i*_ = *n*_*i*0_ + *n*_*i*1_, *n*_*i*0_ = #{*j*:*Y*_*ij*_ = 0} and *n*_*i*1_ = #{*j*:*Y*_*ij*_ > 0}; *j* = 1, 2, …, *n*_*i*_. For *Y*_*i*_ > 0, *W*_*i*_ = ln*Y*_*i*_ are normally distributed with mean *μ*_*i*_ and variance ${\sigma }_{i}^{2}$. For a ZILN model, the maximum likelihood estimates of *d*_*i*_, *μ*_*i*_ and ${\sigma }_{i}^{2}$ are ${\hat {d}}_{i}={n}_{i0}/{n}_{i}$, ${\hat {\mu }}_{i}={\sum }_{j:{Y}_{ij}\gt 0}\ln {Y}_{ij}/{n}_{i1}$ and ${\hat {\sigma }}_{i,mle}^{2}={\sum }_{j:{Y}_{ij}\gt 0}[\ln {Y}_{ij}-{\hat {\mu }}_{i}]^{2}/{n}_{i1}$, respectively. For the *i*th group, the population variance of *Y*_*i*_ is given by

(2) \begin{eqnarray*}{V}_{i}={d}_{i}^{{^{\prime}}}\exp \nolimits (2{\mu }_{i}+{\sigma }_{i}^{2})[\exp \nolimits ({\sigma }_{i}^{2})-{d}_{i}^{{^{\prime}}}]\end{eqnarray*}

which can be log-transformed as ${T}_{i}=\ln {V}_{i}=\ln {d}_{i}^{{^{\prime}}}+2({\mu }_{i}+{\sigma }_{i}^{2})+\ln [1- \frac{{d}_{i}^{{^{\prime}}}}{\exp ({\sigma }_{i}^{2})} ]$. Considering the third term of *T*_*i*_ leads to obtaining $\text{lim}_{{\sigma }_{i}^{2}\rightarrow \infty }\ln [1- \frac{{d}_{i}^{{^{\prime}}}}{\exp ({\sigma }_{i}^{2})} ]=0$ when ${\sigma }_{i}^{2}$ is large. Thus, the log-transformed variance of *V*_*i*_ can be approximated as

(3) \begin{eqnarray*}{T}_{i}\approx \ln \nolimits {d}_{i}^{{^{\prime}}}+2({\mu }_{i}+{\sigma }_{i}^{2}).\end{eqnarray*}

Given ${\hat {d}}_{i}$, ${\hat {\mu }}_{i}$ and ${\hat {\sigma }}_{i}^{2}$ from the observations, the estimates of *T*_*i*_ can be written as ${\hat {T}}_{i}\approx \ln {\hat {d}}_{i}^{{^{\prime}}}+2({\hat {\mu }}_{i}+{\hat {\sigma }}_{i}^{2})$; ${\hat {\sigma }}_{i}^{2}={\sum }_{j:{y}_{ij}\gt 0}[\ln {Y}_{ij}-{\hat {\mu }}_{i}]^{2}/({n}_{i1}-1)$. Using the delta theorem, the variance of ${\hat {T}}_{i}$ becomes

(4) \begin{eqnarray*}\text{Var}({\hat {T}}_{i})= \frac{1-{d}_{i}^{{^{\prime}}}}{{n}_{i}{d}_{i}^{{^{\prime}}}} +4 \left( \frac{{\sigma }_{i}^{2}}{{n}_{i1}} + \frac{2{\sigma }_{i}^{2}}{{n}_{i1}-1} \right) .\end{eqnarray*}

In the present study, the parameter of interest is all pairwise ratios among the log-transformed variances of several ZILN models, which is defined as

(5) \begin{eqnarray*}{\lambda }_{ik}=\ln \nolimits \left( \frac{{V}_{i}}{{V}_{k}} \right) ={T}_{i}-{T}_{k}.\end{eqnarray*}

Its estimates can be obtained as ${\hat {\lambda }}_{ik}={\hat {T}}_{i}-{\hat {T}}_{k}$; ∀*i* ≠ *k* and *i*, k =1 , 2, …, *h*. From Eq. (4), the variance of ${\hat {\lambda }}_{ik}$ can be expressed as

(6) \begin{eqnarray*}\text{Var}({\hat {\lambda }}_{ik})=\text{Var}({\hat {T}}_{i})+\text{Var}({\hat {T}}_{k}),\end{eqnarray*}

where the covariance between ${\hat {T}}_{i}$ and ${\hat {T}}_{k}$ is $\text{COV}({\hat {T}}_{i},{\hat {T}}_{k})=0$ because *Y*_*i*_ = (*Y*_*i*1_, *Y*_*i*2_, …., *Y*_*in*_*i*__) comprise independent and identically distributed (iid) random vector from a ZILN model. Thus, we can obtain estimates of ${\hat {T}}_{i}$ that are independent random variables. Using estimates $({\hat {d}}_{i}^{{^{\prime}}},{\hat {\mu }}_{i},{\hat {\sigma }}_{i}^{2})$ and ${\hat {d}}_{k}^{{^{\prime}}},{\hat {\mu }}_{k},{\hat {\sigma }}_{k}^{2}$ from the samples enables the estimated variance of ${\hat {\lambda }}_{ik}$ to become

(7) \begin{eqnarray*}\widehat{Var}({\hat {\lambda }}_{ik})& =& \frac{1-{\hat {d}}_{i}^{{^{\prime}}}}{{n}_{i}{\hat {d}}_{i}^{{^{\prime}}}} + \frac{1-{\hat {d}}_{i}^{{^{\prime}}}}{{n}_{k}{\hat {d}}_{k}^{{^{\prime}}}} +4 \left( \frac{{\hat {\sigma }}_{i}^{2}}{{n}_{i1}} + \frac{2{\hat {\sigma }}_{i}^{2}}{{n}_{i1}-1} + \frac{{\hat {\sigma }}_{k}^{2}}{{n}_{k1}} + \frac{2{\hat {\sigma }}_{k}^{2}}{{n}_{k1}-1} \right) ,\end{eqnarray*}

where $({\hat {d}}_{i}^{{^{\prime}}},{\hat {\mu }}_{i},{\hat {\sigma }}_{i}^{2})$ and $({\hat {d}}_{k}^{{^{\prime}}},{\hat {\mu }}_{k},{\hat {\sigma }}_{k}^{2})$ denote the estimated parameters of $({d}_{i}^{{^{\prime}}},{\mu }_{i},{\sigma }_{i}^{2})$ and $({d}_{k}^{{^{\prime}}},{\mu }_{k},{\sigma }_{k}^{2})$, respectively.

### Methods

To estimate *λ*_*ik*_, the SCIs are formulated based on Bayesian, GPQ and PB approaches.

#### The Bayesian approach

The essential feature of Bayesian approach is to use the situation-specific prior distribution that reflects knowledge or subjective belief about the parameter of interest; this is modified in accordance with Baye’s Theorem to yield the posterior distribution. Thus, CIs based on the Bayesian approach are derived by using the posterior distribution. In Bayesian theory, the CI is referred to as the credible interval because it is not unique on the posterior distribution. The following methods are used to define suitable credible intervals: the narrowest interval for a univariate distribution (the highest posterior density interval) (Box & Tiao, 1973); the interval when the probability of being below is the same as being above, which is sometimes referred to as the equal-tailed interval (Gelman et al., 2014); or the interval with the mean as the central point (assuming that it exists). In the present study, the SCIs based on the Bayesian approach were constructed based on the equal-tailed interval. Motivated by Maneerat, Niwitpong & Niwitpong (2020), the probability-matching-beta (PMB) and reference-beta (RB) priors were our choice for parameter $({d}_{i}^{{^{\prime}}},{\mu }_{i},{\sigma }_{i}^{2})$ in this study. Thus, Bayesian SCIs for *λ*_*ik*_ were established as follows:

*The PMB prior:*

The probability-matching prior for $({\mu }_{i},{\sigma }_{i}^{2})$ is $\text{P}({\mu }_{i},{\sigma }_{i}^{2})_{pm}\propto {\sigma }_{i}^{-2}\sqrt{2+{\sigma }_{i}^{-2}}$ combined with the prior of ${d}_{i}^{{^{\prime}}}$ as a beta distribution with *a*_*i*_ = *b*_*i*_ = 1/2. Thus, the PMB prior for $({d}^{{^{\prime}}},{\mu }_{i},{\sigma }_{i}^{2})$ can be defined as

(8) \begin{eqnarray*}\text{P}({d}^{{^{\prime}}},{\mu }_{i},{\sigma }_{i}^{2})_{\text{pmb}}\propto \prod _{i=1}^{h}{\sigma }_{i}^{-2}\sqrt{ \frac{2+{\sigma }_{i}^{-2}}{(1-{d}_{i}^{{^{\prime}}}){d}_{i}^{{^{\prime}}}} }.\end{eqnarray*}

When updated with its likelihood, we obtain

(9) \begin{eqnarray*}\text{P}(y{|}\lambda )\propto \prod _{i=1}^{h}(1-{d}_{i}^{{^{\prime}}})_{i}^{{n}_{i0}}{d}_{i}^{{n}_{i1}}({\sigma }_{i}^{2})^{-{n}_{i1}/2}\exp \nolimits \left\{ - \frac{1}{2{\sigma }_{i}^{2}} \sum _{j:{x}_{ij}\gt 0}{ \left( \ln \nolimits {y}_{ij}-{\mu }_{i} \right) }^{2} \right\} .\end{eqnarray*}

The respective marginal posterior distributions of $({d}_{i}^{{^{\prime}}},{\mu }_{i},{\sigma }_{i}^{2})$ are

(10) \begin{eqnarray*}\text{P}({d}_{i}^{{^{\prime}}}{|}{y}_{i})_{\text{pmb}}& \propto & (1-{d}_{i}^{{^{\prime}}})_{i}^{{n}_{i0}+1/2}{d}_{i}^{{n}_{i1}+1/2}\end{eqnarray*}

(11) \begin{eqnarray*}\text{P}({\mu }_{i}{|}{y}_{i},{\sigma }_{i}^{2})_{\text{pmb}}& \propto & \exp \nolimits \left\{ - \frac{1}{2{\sigma }_{i,\text{pmb}}^{2}} \sum _{j:{x}_{ij}\gt 0}{ \left( \ln \nolimits {y}_{ij}-{\mu }_{i} \right) }^{2} \right\} \end{eqnarray*}

(12) \begin{eqnarray*}\text{P}({\sigma }_{i}^{2}{|}{y}_{i})_{\text{pmb}}& \propto & ({\sigma }_{i}^{2})^{- \frac{{n}_{i1}+1}{2} }\sqrt{2+{\sigma }_{i}^{-2}}\exp \nolimits \left\{ - \frac{({n}_{i1}-1){\hat {\sigma }}_{i}^{2}}{2{\sigma }_{i}^{2}} \right\} \end{eqnarray*}

which are denoted as ${d}_{i,\text{pmb}}^{(post)}{|}{y}_{i}\sim \text{beta}({n}_{i0}+1/2,{n}_{i1}+1/2)$, ${\mu }_{i,\text{pmb}}^{(post)}{|}{y}_{i}\sim \text{N}({\hat {\mu }}_{i,\text{pmb}},{\sigma }_{i,\text{pmb}}^{2(post)})$, and ${\sigma }_{i,\text{pmb}}^{2(post)}\propto ({\sigma }_{i}^{2})^{- \frac{{n}_{i1}+1}{2} }\sqrt{2+{\sigma }_{i}^{-2}}\exp \left\{ - \frac{({n}_{i1}-1){\hat {\sigma }}_{i}^{2}}{2{\sigma }_{i}^{2}} \right\} $, respectively. Thus, the posterior of *λ* becomes

(13) \begin{eqnarray*}{\lambda }_{ik,\text{pmb}}^{(post)}={T}_{i,\text{pmb}}^{(post)}-{T}_{k,\text{pmb}}^{(post)},\end{eqnarray*}

where ${T}_{i,\text{pmb}}^{(post)}\approx \ln {d}_{i,\text{pmb}}^{(post)}+2({\mu }_{i}^{(post)}+{\sigma }_{i,\text{pmb}}^{2(post)})$ and ${T}_{k,\text{pmb}}^{(post)}\approx \ln {d}_{k,\text{pmb}}^{(post)}+2({\mu }_{k,\text{pmb}}^{(post)}+{\sigma }_{k,\text{pmb}}^{2(post)})$. In agreement with Ganesh (2009), the 100(1 − *α*)% Bayesian-based SCI with PMB prior for *λ*_*ik*_ is

(14) \begin{eqnarray*}[{L}_{{\lambda }_{ik}},{U}_{{\lambda }_{ik}}]_{\text{pmb}}= \left[ {\lambda }_{ik,\text{pmb}}^{(post)}\mp {v}_{\alpha }^{\text{pmb}} \right] ,\end{eqnarray*}

where ${v}_{\alpha }^{\text{pmb}}$ stands for the (1 − *α*)^*th*^ percentile of the distribution of ${V}^{\text{pmb}}=\text{max}_{h} \left\{ {\lambda }_{ik,\text{pmb}}^{(post)} \right\} -\text{min}_{h} \left\{ {\lambda }_{ik,\text{pmb}}^{(post)} \right\} $.

*The RB prior:*

This is a non-informative prior derived from the Fisher information matrix (Maneerat, Niwitpong & Niwitpong, 2020). The RB prior of $({d}^{{^{\prime}}},{\mu }_{i},{\sigma }_{i}^{2})$ is defined as

(15) \begin{eqnarray*}\text{P}({d}^{{^{\prime}}},{\mu }_{i},{\sigma }_{i}^{2})_{\text{rfb}}\propto \prod _{i=1}^{h}{\sigma }_{i}^{-1}\sqrt{ \frac{1+(2{\sigma }_{i}^{2})^{-1}}{(1-{d}_{i}^{{^{\prime}}}){d}_{i}^{{^{\prime}}}} }\end{eqnarray*}

in which the prior of *d*′ is a beta distribution. When combined with its likelihood Eq. (9), the posterior of $({\mu }_{i},{\sigma }_{i}^{2})$ differs from the PMB prior as follows:

(16) \begin{eqnarray*}\text{P}({\mu }_{i}{|}{y}_{i},{\sigma }_{i}^{2})_{\text{rfb}}& \propto & \exp \nolimits \left\{ - \frac{1}{2{\sigma }_{i,\text{rfb}}^{2}} \sum _{j:{x}_{ij}\gt 0}{ \left( \ln \nolimits {y}_{ij}-{\mu }_{i} \right) }^{2} \right\} \end{eqnarray*}

(17) \begin{eqnarray*}\text{P}({\sigma }_{i}^{2}{|}{y}_{i})_{\text{rfb}}& \propto & ({\sigma }_{i}^{2})^{- \frac{{n}_{i1}}{2} }\sqrt{1+(2{\sigma }_{i}^{2})^{-1}}\exp \nolimits \left\{ - \frac{({n}_{i1}-1){\hat {\sigma }}_{i}^{2}}{2{\sigma }_{i}^{2}} \right\} .\end{eqnarray*}

Moreover, it can be similarly denoted as ${d}_{i,\text{rfb}}^{(post)}{|}{y}_{i}\sim \text{beta}({n}_{i0}+1/2,{n}_{i1}+1/2)$, ${\mu }_{i,\text{rfb}}^{(post)}{|}{y}_{i}\sim \text{N}({\hat {\mu }}_{i,\text{rfb}},{\sigma }_{i,\text{rfb}}^{2(post)})$ and ${\sigma }_{i,\text{rfb}}^{2(post)}\propto ({\sigma }_{i}^{2})^{- \frac{{n}_{i1}}{2} }\sqrt{1+(2{\sigma }_{i}^{2})^{-1}}\exp \left\{ - \frac{({n}_{i1}-1){\hat {\sigma }}_{i}^{2}}{2{\sigma }_{i}^{2}} \right\} $, respectively. The posterior of *λ*_*ik*_ is ${\lambda }_{ik,\text{rfb}}^{(post)}={T}_{i,\text{rfb}}^{(post)}-{T}_{k,\text{rfb}}^{(post)}$, where ${T}_{i,\text{rfb}}^{(post)}\approx \ln {d}_{i,\text{rfb}}^{(post)}+2({\mu }_{i}^{(post)}+{\sigma }_{i,\text{rfb}}^{2(post)})$ and ${T}_{k,\text{rfb}}^{(post)}\approx \ln {d}_{k,\text{rfb}}^{(post)}+2({\mu }_{k,\text{rfb}}^{(post)}+{\sigma }_{k,\text{rfb}}^{2(post)})$. According to Ganesh (2009), the 100(1 − *α*)% Bayesian-based SCI with the RB prior for *λ*_*ik*_ is

(18) \begin{eqnarray*}[{L}_{{\lambda }_{ik}},{U}_{{\lambda }_{ik}}]_{\text{rfb}}= \left[ {\lambda }_{ik,\text{rfb}}^{(post)}\mp {v}_{\alpha }^{\text{rfb}} \right] ,\end{eqnarray*}

where ${v}_{\alpha }^{\text{rfb}}$ stands for the (1 − *α*)^*th*^ percentile of the distribution of ${V}^{\text{rfb}}=\text{max}_{h} \left\{ {\lambda }_{ik,\text{rfb}}^{(post)} \right\} -\text{min}_{h} \left\{ {\lambda }_{ik,\text{rfb}}^{(post)} \right\} $.

#### The GPQ approach

Motivated by Wu & Hsieh (2014), the GPQ of *d*_*i*_ is formulated using the arcsin square-root transformation of the variance. Moreover, the GPQs for $({\mu }_{i},{\sigma }_{i}^{2})$ are also obtained from transformation of the normal approximation by using the central limit theorem (Tian, 2005; Hasan & Krishnamoorthy, 2017). The GPQ for *T*_*i*_ can be written as

(19) \begin{eqnarray*}{G}_{{T}_{i}}=\ln \nolimits \left[ 1-{\sin \nolimits }^{2} \left\{ {\sin \nolimits }^{-1}\sqrt{{\hat {d}}_{i}}- \frac{{R}_{i}}{2\sqrt{{n}_{i}}} \right\} \right] +2 \left[ {\hat {\mu }}_{i}-{S}_{i}\sqrt{ \frac{{\text{G}}_{{\sigma }_{i}^{2}}}{{n}_{i1}} }+{\text{G}}_{{\sigma }_{i}^{2}} \right] ,\end{eqnarray*}

where ${\text{G}}_{{\sigma }_{i}^{2}}=({n}_{i1}-1){\hat {\sigma }}_{i}^{2}/{U}_{i}$. The random variables ${R}_{i}=2\sqrt{{n}_{i}} \left( {\sin }^{-1}\sqrt{{\hat {d}}_{i}}-{\sin }^{-1}\sqrt{{d}_{i}} \right) $, ${S}_{i}=({\hat {\mu }}_{i}-{\mu }_{i})/({\sigma }_{i}^{2}/{n}_{i1})$ and ${U}_{i}=({n}_{i1}-1){\hat {\sigma }}_{i}^{2}/{\sigma }_{i}^{2}$ are independent from standard normal, normal and ${\chi }_{{n}_{i1-1}}^{2}$ distributions, respectively. Thus, the corresponding GPQ of *λ*_*ik*_ can be expressed as

(20) \begin{eqnarray*}{\text{G}}_{{\lambda }_{ik}}={\text{G}}_{{T}_{i}}-{\text{G}}_{{T}_{k}}.\end{eqnarray*}

Similarly, ${\text{G}}_{{T}_{k}}=\ln (1-{\text{G}}_{{d}_{k}})+{\text{G}}_{2{\mu }_{k}}+{\text{G}}_{2{\sigma }_{k}^{2}}$ denotes the GPQ of *T*_*k*_; ${\text{G}}_{{d}_{k}}={\sin }^{2} \left\{ {\sin }^{-1}\sqrt{{\hat {d}}_{k}}- \left[ {R}_{k}{ \left( 2\sqrt{{n}_{k}} \right) }^{-1} \right] \right\} $, ${\text{G}}_{2{\mu }_{k}}=2 \left( {\hat {\mu }}_{k}-{S}_{k}\sqrt{{\text{G}}_{{\sigma }_{k}^{2}}/{n}_{k1}} \right) $, and ${\text{G}}_{2{\sigma }_{k}^{2}}=2({n}_{k1}-1){\hat {\sigma }}_{k}^{2}/{U}_{k}$. Therefore, the 100(1 − *α*)% SCI for *λ*_*jk*_ based on the GPQ approach is given by

(21) \begin{eqnarray*}[{L}_{{\lambda }_{ik}},{U}_{{\lambda }_{ik}}]_{\text{gpq}}= \left[ {\hat {\lambda }}_{ik}\mp {q}_{\alpha }^{\text{GPQ}}\sqrt{\widehat{Var}({\hat {\lambda }}_{ik})} \right] ,\end{eqnarray*}

where ${q}_{\alpha }^{\text{GPQ}}$ denotes the (1 − *α*)^*th*^ percentile of the *Q*^GPQ^ distribution; the *Q*^GPQ^ is derived as

(22) \begin{eqnarray*}{Q}^{\text{GPQ}}=max_{j\not = l} \left\vert \left\{ {\hat {\lambda }}_{ik}-{\text{G}}_{{\lambda }_{ik}}(Y,{Y}^{\ast },d,\mu ,{\sigma }^{2}) \right\} /\sqrt{\widehat{Var}({\hat {\lambda }}_{ik})} \right\vert .\end{eqnarray*}

In agreement with Hannig et al. (2006), Kharrati-Kopaei & Eftekhar (2017), the asymptotic coverage probability of the SCI for *λ*_*ik*_ based on the GPQ is slightly modified from that in Maneerat, Niwitpong & Niwitpong (2021b) (the proof of Theorem 1 in the Appendix).

Theorem 1 *Let* ${Y}_{i}=({Y}_{i1},{Y}_{i2},\ldots .,{Y}_{i{n}_{i}})\sim ^{iid}ZILN({d}_{i},{\mu }_{i},{\sigma }_{i}^{2})$*. For Y*_*i*_ = 0, *n*_*i*0_ *is binomially distributed with the proportion of zero inflation d*_*i*_ = *E*(*n*_*i*0_/*n*_*i*_) *. For Y*_*i*_ > 0, ln*Y*_*i*_ *is log-normally distributed with mean μ*_*i*_ = *E*(ln*Y*_*i*_)*and variance* ${\sigma }_{i}^{2}=Var(\ln {Y}_{i})$*. Moreover, let λ*_*ik*_ = *T*_*i*_/*T*_*k*_; ${T}_{i}\approx \ln {d}_{i}+2({\mu }_{i}+{\sigma }_{i}^{2})$*from group ibe the log-transformed variance of ZILN. Given y*_*i*_ = (*y*_*i*1_, *y*_*i*2_, …., *y*_*in*_*i*__)*, let* $\widehat{Var}({\hat {\lambda }}_{ik})$*be an approximated variance of* ${\hat {\lambda }}_{ik}={\hat {T}}_{i}/{\hat {T}}_{k}$*, where* $({\hat {T}}_{i},{\hat {T}}_{k})$ *are the estimates of* (*T*_*i*_, *T*_*k*_)*. Suppose that n*_*i*_/*n* → *φ*_*i*_ ∈ (0, 1) *as* $n={\mathop{\sum }\nolimits }_{i=1}^{h}{n}_{i}\rightarrow \infty $*, thus it follows that the asymptotically coverage probability of 100* (1 − *α*)% *SCI for λ*_*jk*_ *based the GPQ approach is given by*

(23) \begin{eqnarray*}\mathrm{P} \left( {\lambda }_{jk}\in \left[ {\hat {\lambda }}_{ik}\mp {q}_{\alpha }^{\mathrm{GPQ}}\sqrt{\widehat{Var}({\hat {\lambda }}_{ik})} \right] \right) \rightarrow 1-\alpha \end{eqnarray*}

for ∀*i* ≠ *k and i*, *k* =*1* , …, *h*.

#### The PB approach

Here, we assume that the data come from a known distribution with unknown parameters that are estimated by using samples stimulated from the estimated distribution. In the present study, the PB approach is adjusted to suit our particular situation. Let ${\hat {d}}_{i}^{\ast }$, ${\hat {\mu }}_{i}^{\ast }$ and ${\hat {\sigma }}_{i}^{2\ast }$ be the observed values of ${\hat {d}}_{i}$, ${\hat {\mu }}_{i}$, and ${\hat {\sigma }}_{i}^{2}$ representing the estimated values of parameters *d*_*i*_, *μ*_*i*_, and ${\sigma }_{i}^{2}$, respectively. Thus, we can obtain the empirical distribution of *T* based on the PB approach. In accordance with Sadooghi-Alvandi & Malekzadeh (2014), the respective sampling distributions of (${\hat {d}}_{i}^{\ast }$, ${\hat {\mu }}_{i}^{\ast }$, ${\hat {\sigma }}_{i}^{2\ast }$) are

(24) \begin{eqnarray*}{\hat {d}}_{i}^{(pboot)}& \sim & \text{beta}({n}_{i0}^{\ast }+1/2,{n}_{i1}^{\ast }+1/2)\end{eqnarray*}

(25) \begin{eqnarray*}{\hat {\mu }}_{i}^{(pboot)}& =& {\hat {\mu }}_{i}^{\ast }+{D}_{i}\sqrt{ \frac{{\hat {\sigma }}_{i}^{2\ast }}{{n}_{i1}^{\ast }} }\end{eqnarray*}

(26) \begin{eqnarray*}{\hat {\sigma }}_{i}^{2(pboot)}& =& \frac{{\hat {\sigma }}_{i}^{2\ast }U}{{n}_{i1}^{\ast }-1} ,\end{eqnarray*}

where ${D}_{i}=[{\hat {\mu }}_{i}^{(pboot)}-{\hat {\mu }}_{i}^{\ast }]/\sqrt{{\hat {\sigma }}_{j}^{2\ast }/{n}_{i1}^{\ast }}\sim N(0,1)$ and ${U}_{j}=[{n}_{i1}^{\ast }-1]{\hat {\sigma }}_{i}^{2(pboot)}/{\hat {\sigma }}_{i}^{2\ast }$ $\sim {\chi }_{{n}_{i1}^{\ast }-1}^{2}$ are independent random variables with standard normal and Chi-square distributions, respectively. The PB variable-based pivotal quantity is expressed as

(27) \begin{eqnarray*}{M}^{\text{PB}}= \left\vert \left( {\hat {\lambda }}_{ik}^{pboot}-{\hat {\lambda }}_{ik}^{\ast } \right) /\sqrt{\widehat{Var}({\hat {\lambda }}_{ik}^{\ast })} \right\vert ,\end{eqnarray*}

where ${\hat {\lambda }}_{ik}^{(pboot)}={\hat {T}}_{i}^{(pboot)}-{\hat {T}}_{k}^{(pboot)}$ and ${\hat {\lambda }}_{ik}^{\ast }={\hat {T}}_{i}^{\ast }-{\hat {T}}_{k}^{\ast }$. By replacing observed values $({\hat {d}}_{i}^{\ast }{\hat {\mu }}_{i}^{\ast },{\hat {\sigma }}_{i}^{2\ast })$ from the samples, we respectively obtain

(28) \begin{eqnarray*}{\hat {\lambda }}_{ik}^{\ast }& =& \ln \nolimits \left( \frac{{\hat {d}}_{i}^{\ast }}{{\hat {d}}_{k}^{\ast }} \right) +2 \left[ ({\hat {\mu }}_{i}^{\ast }-{\hat {\mu }}_{k}^{\ast })+({\hat {\sigma }}_{i}^{2\ast }-{\hat {\sigma }}_{k}^{2\ast }) \right] \end{eqnarray*}

(29) \begin{eqnarray*}{\hat {\lambda }}_{ik}^{(pboot)}& =& \ln \nolimits \left( \frac{{\hat {d}}_{i}^{(pboot)}}{{\hat {d}}_{k}^{(pboot)}} \right) +2 \left[ ({\hat {\mu }}_{i}^{(pboot)}-{\hat {\mu }}_{k}^{(pboot)})+({\hat {\sigma }}_{i}^{2(pboot)}-{\hat {\sigma }}_{k}^{2(pboot)}) \right] \end{eqnarray*}

(30) \begin{eqnarray*}\hat {Var}({\hat {\lambda }}_{ik}^{\ast })& =& \frac{{\hat {d}}_{i}^{\ast }}{{n}_{i}{\hat {d}}_{i}^{\ast }} + \frac{{\hat {d}}_{i}^{\ast }}{{n}_{k}{\hat {d}}_{k}^{\ast }} +4 \left( \frac{{\hat {\sigma }}_{i}^{2\ast }}{{n}_{i1}^{\ast }} + \frac{2{\hat {\sigma }}_{i}^{2\ast }}{{n}_{i1}^{\ast }-1} + \frac{{\hat {\sigma }}_{k}^{2\ast }}{{n}_{k1}^{\ast }} + \frac{2{\hat {\sigma }}_{k}^{2\ast }}{{n}_{k1}^{\ast }-1} \right) ,\end{eqnarray*}

where ${\hat {d}}_{i}^{\ast }=1-{\hat {d}}_{i}^{\ast }$ and ${n}_{i1}^{\ast }={n}_{i}{\hat {d}}_{i}^{\ast }$. Hence, the 100(1 − *α*)% SCI for *λ*_*ik*_ based on the PB approach is

(31) \begin{eqnarray*}[{L}_{{\lambda }_{ik}},{U}_{{\lambda }_{ik}}]_{(PB)}= \left[ {\hat {\lambda }}_{ik}\mp {M}_{\alpha }^{\text{PB}}\sqrt{\widehat{Var}({\hat {\lambda }}_{ik})} \right] ,\end{eqnarray*}

where ${m}_{\alpha }^{\text{PB}}$ is the (1 − *α*)^*th*^ percentile of the distribution of *M*^PB^. Theorem 2 shows the asymptotic coverage probability of the 100(1 − *α*)% SCI for *λ*_*ik*_ based on the PB approach (see the proof in the Appendix ).

Theorem 2 *Suppose that Y*_*i*_ = (*Y*_*i*1_, *Y*_*i*2_, …., *Y*_*in*_*i*__) *comprise an iid random vector from a ZILN model based on n*_*i*_ *observations from population group i. Let* ${\hat {\lambda }}_{ik}={\hat {T}}_{i}-{\hat {T}}_{k}$*be the estimate of λ*_*ik*_*, where* ${\hat {T}}_{i}$ *and* ${\hat {T}}_{k}$ *are the approximately log-transformed variances of* ${\hat {T}}_{i}$* and* ${\hat {T}}_{k}$ *from the population groups ith and kth, respectively. Hence,*

(32) \begin{eqnarray*}\mathrm{P} \left[ {\lambda }_{ik}\in \left( {\hat {\lambda }}_{ik}\mp {M}_{\alpha }^{\mathrm{PB}}\sqrt{\widehat{Var}({\hat {\lambda }}_{ik})} \right) \right] \rightarrow 1-\alpha ,\end{eqnarray*}

*where* $\widehat{Var}({\hat {\lambda }}_{ik})$*is the estimated variance of* ${\hat {\lambda }}_{ik}$*;* ∀*i* ≠ *k and i*, *k* =*1* , 2, .., *h*.

## Simulation studies and results

Simulation studies were conducted to assess the performances of the SCIs based Bayesian, GPQ, and PB approaches for all pairwise ratios of variances of several ZILN distributions: Bayesian SCIs based on PMB and RB priors (Maneerat, Niwitpong & Niwitpong, 2020), the GPQ-based SCI (Wu & Hsieh, 2014), and the PB-based SCI (Sadooghi-Alvandi & Malekzadeh, 2014; Li, Song & Shi, 2015; Kharrati-Kopaei & Eftekhar, 2017). CRs, LERs, UERs, and AWs of the SCIs were determined when the population group size(*h*) were fixed at 3 and 5; the optimal values of CR, LER, UER, and AW are 95%, 5%, 5% and 0, respectively, which were used to judge the best-performing SCI. Critical values ${v}_{\alpha }^{\text{pmb}}$, ${v}_{\alpha }^{\text{rb}}$, ${q}_{\alpha }^{\text{GPQ}}$ and ${m}_{\alpha }^{\text{PB}}$ for the Bayesian SCIs based on PMB and RB priors, GPQ and PB, respectively, were also assessed. Throughout the simulation studies, the simulation procedure to estimate the CRs, LERs, and UERs was as follows:

(i) Generate random samples *Y*_*i*_ = (*Y*_*i*1_, *Y*_*i*2_, …., *Y*_*in*_*i*__) from $\text{ZILN}({d}_{i},{\mu }_{i},{\sigma }_{i}^{2})$, and compute ${\hat {d}}_{i}$, ${\hat {\mu }}_{i},{\hat {\sigma }}_{i}^{2}$; *i* = 1, 2, …, *h* from the samples.
(ii) Compute the critical values for each method using 2500 Monte Carlo simulations.
(iii) Apply the SCIs based on Bayesian-based PMB and RB priors, GPQ, and PB approaches given in Eqs. (14), (18), (21) and (31), respectively, and record whether or not the values of (*λ*_*ik*_; *i* ≠ *k*) fall within their corresponding confidence intervals.
(iv) Repeat steps (i)-(iii) *M* = 5000 times.
(v) For each method: obtain the number of times that all (*λ*_*ik*_; *i* ≠ *k*) are in their corresponding SCIs to estimated the CR.
(vi) Obtain the number of times that all (*λ*_*ik*_; *i* ≠ *k*) is less than or greater than their corresponding SCIs to estimate the LER and UER, respectively.

For the three-group comparison, the following parameter combinations were used: large variances $({\sigma }_{1}^{2},{\sigma }_{2}^{2},{\sigma }_{3}^{2})=(3,5,7)$; small (30, 30, 30), moderate (50, 50, 50), large [(100, 100, 100) and (100, 100, 200)], small-to-large (30, 50, 100) and medium-to-large (50, 100, 200) sample sizes; and zero-inflation percentages of (10, 20, 30), (10, 30, 50) and (30, 50, 50). For the five-group comparison, the following parameter combinations were used: large variances $({\sigma }_{1}^{2},{\sigma }_{2}^{2},{\sigma }_{3}^{2},{\sigma }_{4}^{2},{\sigma }_{5}^{2})$ =(1, 1, 2, 2, 3); small-to-large (30, 50, 50, 100, 200), medium-to-large (50, 50, 50, ) (100, 100), and large (70, 100, 100, 200, 200) sample sizes; and zero-inflation percentages of (10, 10, 20, 20, 20), (20, 20, 30, 30, 50) and (50, 50, 50, 70, 70). The results are reported in Table 1.

**Table 1:** Performance measures of SCIs-based different approaches.

*n*_*i*_	*d*_*i*_(%)	B-PMB			B-RB			GPQ			PB			AW			
		LER	CR	UER	LER	CR	UER	LER	CR	UER	LER	CR	UER	B-PMB	B-RB	GPQ	PB
3 sample groups and $({\sigma }_{1}^{2},{\sigma }_{2}^{2},{\sigma }_{3}^{2})=(3,5,7)$																	
(30_3_)	(10,20,30)	1.993	97.973	0.033	1.307	98.693	0.000	0.707	99.293	0.000	2.460	97.540	0.000	22.961	25.493	27.764	**22.942**
	(10,30,50)	1.880	98.113	0.007	1.200	98.800	0.000	0.967	99.033	0.000	2.900	97.100	0.000	28.702	33.323	33.300	**27.254**
	(30,50,50)	1.120	98.873	0.007	0.427	99.573	0.000	0.620	99.380	0.000	2.520	97.480	0.000	30.764	35.737	36.813	**29.113**
(50_3_)	(10,20,30)	2.833	96.800	0.367	2.300	97.567	0.133	1.107	98.887	0.007	2.347	97.627	0.027	**15.521**	16.544	19.078	16.893
	(10,30,50)	2.887	97.027	0.087	2.173	97.800	0.027	1.253	98.747	0.000	2.607	97.393	0.000	**18.848**	20.654	22.403	19.733
	(30,50,50)	2.087	97.840	0.073	1.413	98.567	0.020	0.973	99.027	0.000	2.320	97.673	0.007	**20.104**	21.996	24.567	21.096
(100_3_)	(10,20,30)	3.480	95.140	1.380	3.200	95.693	1.107	1.273	98.527	0.200	1.960	97.767	0.273	**10.015**	10.325	12.448	11.681
	(10,30,50)	3.660	95.627	0.713	3.200	96.420	0.380	1.427	98.540	0.033	2.087	97.833	0.080	**11.866**	12.410	14.327	13.422
	(30,50,50)	3.220	96.040	0.740	2.780	96.747	0.473	1.167	98.813	0.020	2.073	97.853	0.073	**12.389**	12.948	15.408	14.202
(30,50,100)	(10,20,30)	1.787	96.753	1.460	1.367	97.453	1.180	0.380	99.480	0.140	1.127	98.467	0.407	**12.846**	13.402	16.552	14.152
	(10,30,50)	1.853	97.127	1.020	1.387	97.993	0.620	0.420	99.553	0.027	1.420	98.353	0.227	**14.348**	15.042	18.368	15.604
	(30,50,50)	1.013	97.947	1.040	0.547	98.687	0.767	0.260	99.653	0.087	1.053	98.627	0.320	**16.343**	17.452	20.826	17.181
(50,100,200)	(10,20,30)	2.580	94.773	2.647	2.247	95.293	2.460	0.467	99.047	0.487	0.847	98.307	0.847	**8.637**	8.822	11.261	10.230
	(10,30,50)	2.847	95.073	2.080	2.593	95.560	1.847	0.667	99.093	0.240	1.313	98.140	0.547	**9.522**	9.725	12.334	11.166
	(30,50,50)	2.173	95.880	1.947	1.793	96.533	1.673	0.380	99.380	0.240	1.020	98.460	0.520	**10.618**	10.939	13.751	12.189
(100_2_,200)	(10,20,30)	3.253	94.213	2.533	2.953	94.693	2.353	0.967	98.673	0.360	1.507	97.920	0.573	**7.952**	8.090	10.266	9.647
	(10,30,50)	2.940	95.013	2.047	2.620	95.533	1.847	0.980	98.793	0.227	1.460	98.127	0.413	**8.985**	9.184	11.489	10.773
	(30,50,50)	2.567	95.387	2.047	2.227	96.007	1.767	0.900	98.893	0.207	1.547	98.047	0.407	**9.888**	10.197	12.666	11.709

5 sample groups and $({\sigma }_{1}^{2},{\sigma }_{2}^{2},{\sigma }_{3}^{2},{\sigma }_{4}^{2},{\sigma }_{5}^{2})=(1,1,2,2,3)$																	
(30, 50_2_, 100, 200)	(10,10,20,20,20)	0.326	99.504	0.170	0.232	99.626	0.142	0.344	99.568	0.088	0.756	99.002	0.242	6.224	6.471	6.310	**5.600**
	(20,20,30,30,50)	0.244	99.620	0.136	0.154	99.754	0.092	0.244	99.694	0.062	0.666	99.164	0.170	6.952	7.250	7.067	**6.201**
	(20,30,50,50,70)	0.154	99.738	0.108	0.092	99.828	0.080	0.322	99.642	0.036	0.788	99.084	0.128	8.510	8.971	8.513	**7.369**
	(50,50,50,70,70)	0.062	99.882	0.056	0.026	99.942	0.032	0.116	99.872	0.012	0.426	99.490	0.084	9.572	10.226	9.861	**8.223**
(50_3_, 100_2_)	(10,10,20,20,20)	0.398	99.504	0.098	0.338	99.582	0.080	0.558	99.414	0.028	1.122	98.788	0.090	6.614	6.826	6.557	**5.914**
	(20,20,30,30,50)	0.392	99.512	0.096	0.312	99.618	0.070	0.526	99.448	0.026	1.092	98.810	0.098	7.791	8.100	7.567	**6.768**
	(20,30,50,50,70)	0.358	99.618	0.024	0.244	99.748	0.008	0.582	99.398	0.020	1.196	98.754	0.050	10.067	10.737	9.488	**8.354**
	(50,50,50,70,70)	0.204	99.766	0.030	0.136	99.850	0.014	0.254	99.746	0.000	0.822	99.166	0.012	10.687	11.352	10.571	**9.039**
(70, 100_2_, 200_2_)	(10,10,20,20,20)	0.784	99.038	0.178	0.710	99.140	0.150	0.810	99.120	0.080	1.232	98.640	0.128	4.499	4.565	4.507	**4.237**
	(20,20,30,30,50)	0.666	99.174	0.160	0.580	99.280	0.140	0.620	99.310	0.070	1.058	98.826	0.116	5.218	5.321	5.116	**4.783**
	(20,30,50,50,70)	0.620	99.290	0.090	0.550	99.380	0.060	0.750	99.200	0.060	1.158	98.744	0.098	6.546	6.743	6.202	**5.753**
	(50,50,50,70,70)	0.374	99.548	0.078	0.310	99.630	0.060	0.370	99.600	0.030	0.680	99.258	0.062	6.938	7.139	6.892	**6.249**

**Notes.**

Note: (100_3_, 200_2_) = (100, 100, 100, 200, 200). Bold denotes the best-performing method.

**Figure 1:**
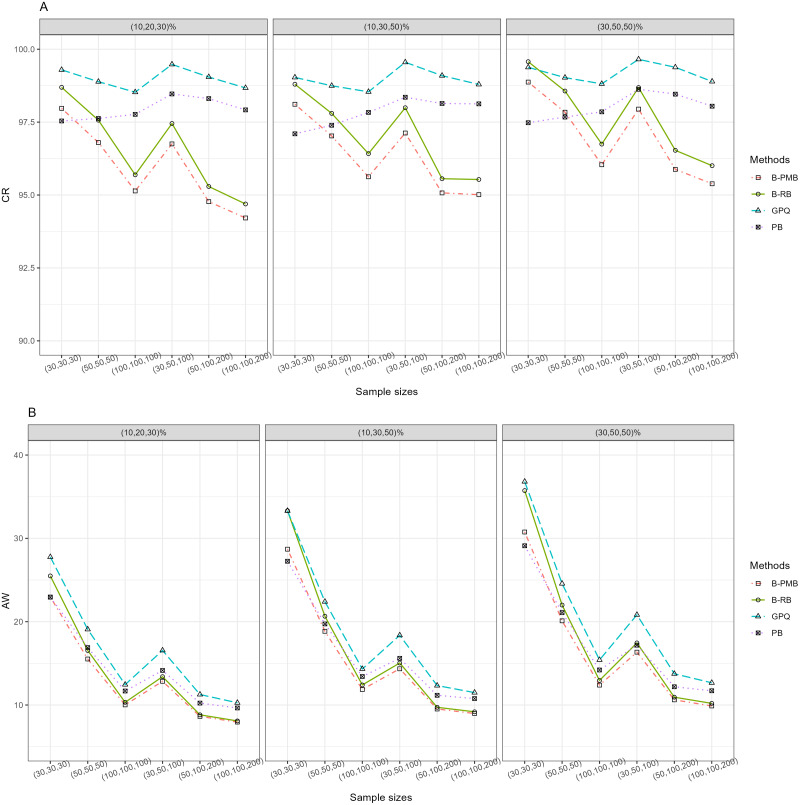
The CR and AW performance measures for three sample groups: (A) CR (B) AW.

**Figure 2:**
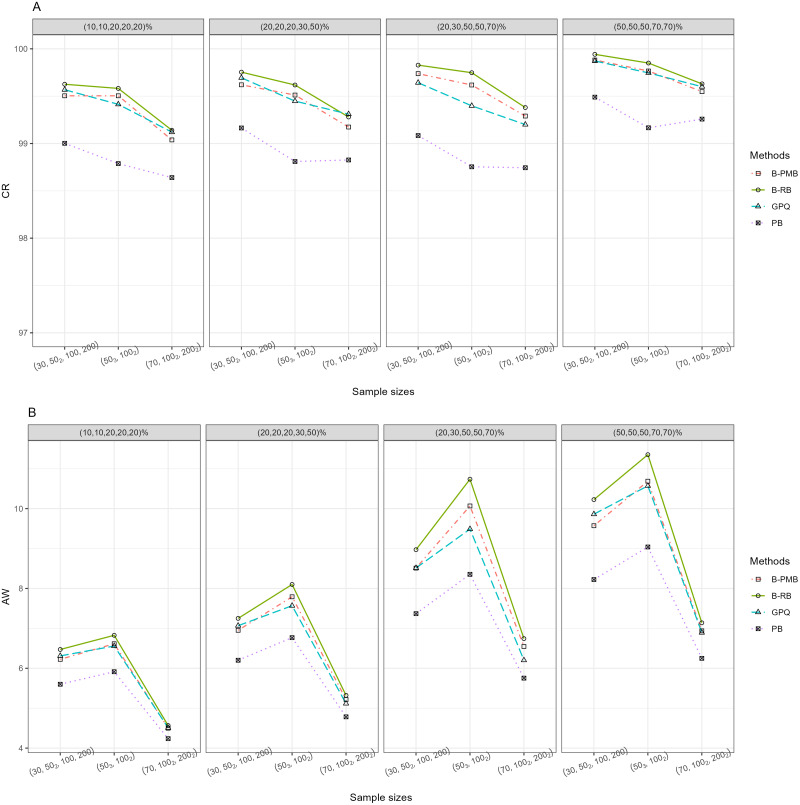
The CR and AW performance measures for five sample groups: (A) CR (B) AW.

For *h* = 3 with large variance, Table 1 and Fig. 1 reveal that all of the methods provided CR performances close to and greater than the nominal confidence level (95%). Meanwhile, the SCIs based on the Bayesian approach based on the PMB prior and GPQ maintained a good balance between LER and UER. Importantly, the AW of PB was narrower than the other methods for small sample sizes, while those of the Bayesian approach based on the PMB prior were slightly narrower than the others for the other sample sizes. When a group comparison was *h* = 5 (Table 1 and Fig. 2), the PB approach provided the best CRs and narrowest AWs for all scenarios tested.

## An empirical application of the four methods to daily precipitation data

Daily precipitation records comprise publicly available data from the Thailand Meteorology Department (Department, 2021). Flash floods, landslides, and windstorms caused by heavy rainfall occurred in the four provinces in the lower southern area of Thailand: Songkhla, Yala, Narathiwat, and Pattani during January 2021, as reported by Thailand’s Department of Disaster Prevention and Mitigation (Thailand, 2021). According to automatic weather system (Department, 2021), Songkhla has two weather stations in the Songkhla and Sadao districts, which means that we could simultaneously estimate variations in precipitation at five weather stations.

**Figure 3:**
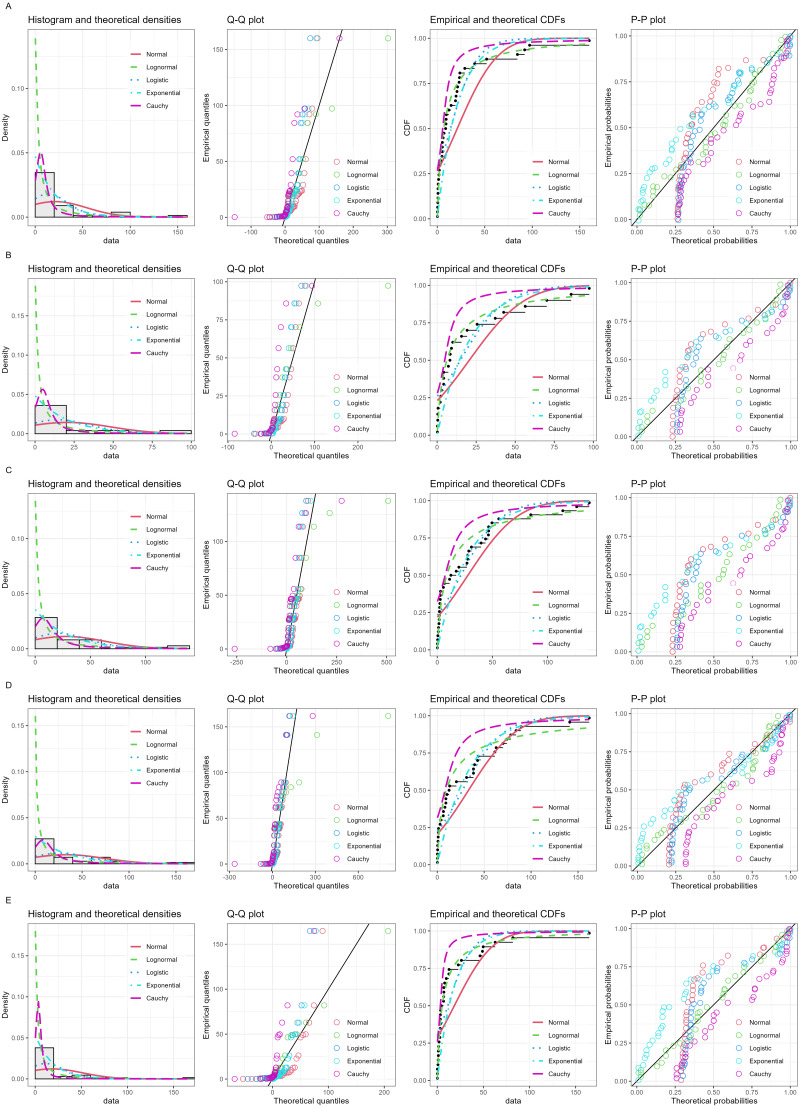
Histogram, normal Q-Q, CDF and P-P plots of nonzero precipitation records in five stations of southern Thailand: (A) Songkhla (B) Songkhla-Sadao (C) Yala (D) Narathiwat (E) Pattani.

Daily precipitation data from December 2020 to January 2021 (Table 2) were used in the analysis. Figure 3 shows histogram along with normal quantile–quantile (Q-Q), cumulative density function (CDF) and probability-probability (P-P) plots. Furthermore, the Akaike information criterion (AIC) and Bayesian information criterion (BIC) values of five models: normal, logistic, lognormal, exponential, and Cauchy applied to fitting the non-zero precipitation data were compared to check the appropriateness of each model for fitting the data (Table 3). The AIC and BIC results for the lognormal model were the lowest, and thus it was the most efficient. The data from all of the stations were zero-inflated, thereby verifying that they follow the assumptions for ZILN.

**Table 2:** Daily precipitation data in five stations of southern Thailand.

Dates	Weather stations: December 2020					Dates	Weather stations: January 2021				
	Shongklha	Songkhla-based Sadao district	Yala	Narathiwat	Pattani		Shongklha	Songkhla-based Sadao district	Yala	Narathiwat	Pattani
1	160.0	56.4	46.4	38.6	82.0	1	0.8	4.2	6.6	31.2	0.8
2	14.6	85.8	46.6	70.0	0.0	2	1.4	8.2	5.6	6.4	2.0
3	20.8	4.2	55.8	74.2	0.0	3	2.6	42.6	49.6	38.6	49.8
4	8.8	0.2	27.0	0.4	7.2	4	21.4	8.4	28.6	10.4	4.4
5	0.0	0.0	0.2	0.0	0.0	5	9.2	70.2	137.8	62.8	49.0
6	0.0	0.0	0.0	0.0	0.2	6	0.2	2.8	84.8	13.2	0.2
7	0.0	0.0	0.0	0.0	0.0	7	0.0	0.0	1.8	9.2	2.8
8	0.2	0.0	1.6	0.0	0.0	8	0.4	0.0	0.4	0.0	1.2
9	52.0	0.0	0.0	0.0	0.0	9	0.8	0.0	0.0	1.4	0.0
10	39.4	0.0	0.0	0.8	3.6	10	29.0	15.6	2.8	12.6	22.8
11	0.6	0.0	2.8	9.2	9.8	11	23.0	0.6	0.2	0.2	0.0
12	12.2	4.2	17.2	0.0	8.0	12	5.0	0.2	0.6	3.6	1.2
13	5.4	37.2	2.0	8.2	12.8	13	0.0	0.0	2.4	3.0	1.0
14	9.4	0.0	0.0	0.0	3.4	14	5.4	0.0	0.0	0.0	0.0
15	7.0	2.4	12.4	78.4	7.2	15	1.8	0.0	0.0	0.0	0.0
16	19.2	25.6	43.8	43.0	62.8	16	0.8	0.0	0.0	0.0	0.0
17	84.4	97.4	126.4	162.0	164.8	17	0.0	0.0	0.0	0.0	0.0
18	97.2	9.2	113.8	141.2	46.4	18	0.0	0.0	0.0	1.2	0.0
19	92.0	19.2	39.8	43.6	26.2	19	0.0	0.0	0.0	0.0	0.0
20	19.8	7.2	27.8	20.4	7.0	20	0.0	0.0	0.0	0.0	0.0
21	5.4	0.4	0.0	0.2	3.4	21	0.0	0.0	0.0	0.0	0.0
22	0.0	0.0	1.2	1.0	3.4	22	0.0	0.0	0.0	0.0	0.0
23	23.8	0.0	31.0	61.4	12.6	23	0.0	0.0	0.0	0.0	0.0
24	23.4	0.0	19.6	6.6	0.0	24	0.0	0.0	2.2	0.0	0.0
25	2.2	0.0	46.6	39.8	6.8	25	0.0	0.0	0.0	0.0	0.0
26	1.0	10.0	27.6	84.0	2.8	26	0.0	0.0	0.0	0.0	0.0
27	0.0	0.0	1.0	0.0	0.2	27	0.0	0.0	2.0	0.2	0.0
28	0.0	0.0	0.0	0.0	0.0	28	0.0	0.0	0.0	0.0	0.0
29	0.0	0.0	0.0	0.0	0.0	29	0.0	0.0	0.0	0.0	0.0
30	0.0	0.0	0.0	0.0	0.0	30	4.4	0.0	0.0	0.0	0.0
31	6.2	0.4	11.2	89.2	3.2	31	9.6	2.0	3.0	0.4	1.6

**Notes.**

Source: Thailand Meteorological Department Automatic Weather System.

http://www.aws-observation.tmd.go.th/web/climate/climate_past.asp.

The results in Table 4 reveals that since variance ${\sigma }_{i}^{2}$ was greater than the mean *μ*_*i*_, quite large precipitation variations were required in the present study. For applying data of daily precipitation to measure the efficacy of the four methods, the 95% SCIs-based Bayesian, GPQ and PB approaches for all pairwise precipitation datasets from the five weather stations cover their point estimates (Table 5). In a agree with the simulation results for *n*_1_ = *n*_2_ = *n*_3_ = 50 and *n*_4_ = *n*_5_ = 100, the PB approach provided the best SCI performance for ratio of variances of several ZILN models. This can be interpreted as Narathiwat has the highest variation in precipitation, followed by Yala. These results are in line with the Asia Disaster Monitoring and Response System (Thailand, 2021), which reported that both areas were affected by flooding and landslides damaging 22,308 households in Narathiwat and 12,082 households in Yala during the time period covered by the data used in the study.

**Table 3:** The AIC and BIC results for five associated models.

Stations	Criterion	Models				
		Normal	Lognormal	Logistic	Exponential	Cauchy
Songkhla	AIC	387.611	305.171	373.337	317.644	345.549
	BIC	390.938	308.498	376.664	319.308	348.876
Songkhla-Sadao district	AIC	241.141	196.707	238.534	203.226	225.198
	BIC	243.579	199.145	240.971	204.445	227.635
Yala	AIC	373.538	313.718	368.171	322.168	365.426
	BIC	376.760	316.940	371.393	323.779	368.648
Narathiwat	AIC	362.209	310.600	359.299	317.455	358.947
	BIC	365.320	313.711	362.410	319.010	362.058
Pattani	AIC	328.067	242.474	313.959	260.584	273.318
	BIC	331.060	245.467	316.952	262.080	276.311

**Table 4:** Summary statistics for five stations.

Weather stations	i	*n*_*i*0_	*n*_*i*1_	${\hat {d}}_{i}$ (%)	${\hat {\mu }}_{i}$	${\sigma }_{i}^{2}$	${\hat {\lambda }}_{i}$
Songkhla	1	39	23	37.097	1.909	2.982	9.317
Songkhla-Sadao district	2	25	37	59.677	1.828	3.509	9.766
Yala	3	37	25	40.323	2.155	3.490	10.774
Narathiwat	4	35	27	43.548	2.253	4.238	12.411
Pattani	5	33	29	46.774	1.669	2.950	8.607

**Table 5:** 95% SCIs of all pairwise log-ratios of precipitation variabilities amoung five weather stations in lower southern Thailand.

Methods	Limits	All pairwise log-ratios of precipitation variabilities among weather stations				
		Songkhla/ Songkhla-sadao	Songkhla/ Yala	Songkhla/ Narathiwat	Songkhla/ Pattani	Songkhla-sadao/Yala
		−0.4489	−1.4568	−3.0939	0.71043	−1.0079
Bayesian SCIs -based PMB prior	Lower	−8.7881	−9.796	−11.4331	−7.6287	−9.3471
	Upper	7.8903	6.8824	5.2452	9.0496	7.3313
	Width	16.6783	16.6783	16.6783	16.6783	16.6783
Bayesian SCIs -based RB prior	Lower	−9.4711	−10.479	−12.1161	−8.3117	−10.0301
	Upper	8.5733	7.5654	5.9283	9.7326	8.0143
	Width	18.0444	18.0444	18.0444	18.0444	18.0444
SCI-based GPQ	Lower	−9.3037	−9.2166	−11.9695	−6.6362	−10.4292
	Upper	8.4059	6.303	5.7816	8.0571	8.4134
	Width	17.7096	15.5196	17.7511	14.6932	18.8426
SCI-based PB	Lower	−7.4257	−7.5709	−10.0871	−5.0781	−8.4311
	Upper	6.5279	4.6573	3.8992	6.4989	6.4153
	Width	13.9536	12.2281	13.9863	11.577	14.8464
Methods	Limits	Songkhla-sadao/ Narathiwat	Songkhla-sadao/ Pattani	Yala/ Narathiwat	Yala/ Pattani	Narathiwat/ Pattani
		−2.645	1.1593	−1.6371	2.1672	3.8043
Bayesian SCIs -based PMB prior	Lower	−10.9842	−7.1798	−9.9763	−6.1719	−4.5348
	Upper	5.6941	9.4985	6.702	10.5064	12.1435
	Width	16.6783	16.6783	16.6783	16.6783	16.6783
Bayesian SCIs -based RB prior	Lower	−11.6672	−7.8629	−10.6593	−6.855	−5.2178
	Upper	6.3771	10.1815	7.385	11.1894	12.8266
	Width	18.0444	18.0444	18.0444	18.0444	18.0444
SCI-based GPQ	Lower	−13.0047	−7.9247	−11.078	−5.8532	−5.2999
	Upper	7.7146	10.2433	7.8037	10.1876	12.9086
	Width	20.7193	18.168	18.8817	16.0408	18.2085
SCI-based PB	Lower	−10.8075	−5.9981	−9.0757	−4.1522	−3.369
	Upper	5.5175	8.3168	5.8014	8.4866	10.9777
	Width	16.325	14.3149	14.8771	12.6388	14.3467

## Discussion

From the above numerical results, it can be seen that the SCIs based on PB and the Bayesian approach based on the PMB prior dealt with large variations in the data better than the other approaches. The PB-based SCI has some strong points for small sample sizes due to random samples being obtained *via* bootstrap resampling. Furthermore, the performance of the Bayesian SCI based on the PMB prior declined as the number of populations increased and the sample size decreased. Although, the GPQ method provided appropriate CRs, its AWs were wider than the other methods, possibly because the GPQ of *d*_*i*_ is limited for cases with unequal zero-inflated percentages. Since it has performed quite well for one population group especially (Wu & Hsieh, 2014; Maneerat, Niwitpong & Niwitpong, 2021a). Further research could be conducted to explore subjective or prior beliefs about parameters when using the Bayesian approach for parameter estimation

## Conclusions

SCIs for the comparison of the variance ratios among several ZILN models were formulated by applying Bayesian approaches based on the PMB and RB priors, along with the GPQ and PB approaches. In practice, the daily precipitation data for each of the weather stations considered were overdispersed (*i.e.,* the variance was greater than the mean) and zero-inflated (Table 4). Thus, the ZILN distribution is an appropriate model for estimating parameters in the construction of SCIs for multiple comparisons between their variances.

For three populations, all of the methods produced 95% SCIs for all pairwise comparisons among variances covering the true parameter. Meanwhile, the SCI constructed *via* the Bayesian approach based on the PMB prior maintained a good balance between LER and UER and provided the narrowest AWs except for small sample sizes. On the other hand, the PB-based SCI could handle extreme cases when the sample sizes were small with large variances. For five populations, the PB-based SCI performed the best overall, with the Bayesian approach based on the RB prior for small-to-large sample sizes and the GPQ approach for medium-to-large and large sample sizes providing acceptable results, and thus can be recommended as alternative SCIs.

## Supplemental Information

10.7717/peerj.12659/supp-1 Supplemental Information 1 R code for computing all results in this paper

## Appendix

The proofs of the methods for constructing the SCI for *λ*_*ik*_ are covered here.

### The GPQ approach

Proof of Theorem 1 The proof is similar to Hannig et al. (2006), Kharrati-Kopaei & Eftekhar (2017), and Maneerat, Niwitpong & Niwitpong (2021b). Since random variable $Q= \frac{{\hat {\lambda }}_{ik}-{\lambda }_{jk}}{\sqrt{\widehat{Var}({\hat {\lambda }}_{ik})}} $ is obtained by applying the central limit theorem, where ${\hat {\lambda }}_{ik}$ is an estimate of the log-ratio transformation of variances of the ZILNs (*λ*_*jk*_), and $\widehat{Var}({\hat {\lambda }}_{ik})$ is the approximate variance of ${\hat {\lambda }}_{ik}$. Consider

(33) \begin{eqnarray*}\mathrm{P} \left( {\lambda }_{jk}\in \left[ {\hat {\lambda }}_{ik}\mp {q}_{\alpha }^{\mathrm{GPQ}}\sqrt{\widehat{Var}({\hat {\lambda }}_{ik})} \right] \right) & =& \text{P} \left( \text{max}_{i\not = k} \left\vert \frac{{\hat {\lambda }}_{ik}-{\lambda }_{jk}}{\sqrt{\widehat{Var}({\hat {\lambda }}_{ik})}} \right\vert \leq {q}_{\alpha }^{\mathrm{GPQ}} \right) \nonumber\\\displaystyle & =& \text{P} \left( {Q}_{n}\leq {q}_{\alpha }^{\text{GPQ}} \right) \end{eqnarray*}

as *n* → ∞. Hence, $\mathrm{P} \left( {\hat {\lambda }}_{ik}-{q}_{\alpha }^{\mathrm{GPQ}}\sqrt{\widehat{Var}({\hat {\lambda }}_{ik})}\lt {\lambda }_{jk}\lt {\hat {\lambda }}_{ik}+ \right. $ $ \left. {q}_{\alpha }^{\mathrm{GPQ}}\sqrt{\widehat{Var}({\hat {\lambda }}_{ik})} \right) \rightarrow 1-\alpha $; ∀*i* ≠ *k*.

### The parametric bootstrap approach

Proof of Theorem 2 The proof is similar to Hannig et al. (2006), Li, Song & Shi (2015); Kharrati-Kopaei & Eftekhar (2017) and Maneerat, Niwitpong & Niwitpong (2021b). Recall that $\widehat{Var}({\hat {\lambda }}_{ik})$ is the estimated variance of ${\hat {\lambda }}_{ik}$. From the 100(1 − *α*)% SCI for *λ*_*ik*_ based on the PB approach, we can derive

(34) \begin{eqnarray*}\mathrm{P} \left[ {\lambda }_{ik}\in \left( {\hat {\lambda }}_{ik}\mp {M}_{\alpha }^{\mathrm{PB}}\sqrt{\widehat{Var}({\hat {\lambda }}_{ik})} \right) \right] =\mathrm{P} \left( \mathrm{max}_{i\not = k} \left\vert \frac{{\hat {\lambda }}_{ik}-{\lambda }_{jk}}{\sqrt{\widehat{Var}({\hat {\lambda }}_{ik})}} \right\vert \leq {m}_{\alpha }^{\mathrm{PB}} \right) \end{eqnarray*}

Let *n*_*i*_/*n* → *φ*_*i*_ as *n* = *n*_1_ + *n*_2_ + ... + *n*_*h*_ → ∞. Thus, by applying the central limit theorem, $n({\hat {\lambda }}_{i}-{\lambda }_{i})\rightarrow {W}_{i}$, we arrive at

(35) \begin{eqnarray*}W=({W}_{1},{W}_{2},\ldots ,{W}_{h})\sim N(0,{\sigma }_{i}^{2}/{\varphi }_{i})\end{eqnarray*}

By applying Slutsky’s theorem, we obtain

(36) \begin{eqnarray*}\mathrm{max}_{i\not = k} \left\vert \frac{{\hat {\lambda }}_{ik}-{\lambda }_{ik}}{\sqrt{\widehat{Var}({\hat {\lambda }}_{ik})}} \right\vert \rightarrow ^{d}\mathrm{max}_{i\not = k} \left\vert \frac{{W}_{i}-{W}_{k}}{\sqrt{ \frac{{\sigma }_{i}^{2}}{{\varphi }_{i}} + \frac{{\sigma }_{k}^{2}}{{\varphi }_{k}} }} \right\vert \end{eqnarray*}

Assume that *W*_*i*_ and ${W}_{i}^{\ast }$ are iid random variables, then

(37) \begin{eqnarray*}T(\mathbf{Y },{\mathbf{Y }}^{\ast },\mathbf{d},\mu ,{\sigma }^{\mathbf{2}})\rightarrow \mathrm{max}_{i\not = k} \left\vert \frac{{W}_{i}^{\ast }-{W}_{k}^{\ast }}{\sqrt{ \frac{{\sigma }_{i}^{2}}{{\varphi }_{i}} + \frac{{\sigma }_{k}^{2}}{{\varphi }_{k}} }} \right\vert ,\end{eqnarray*}

where *T*(**Y**, **Y**^∗^, **d**, *μ*, *σ*^**2**^) comprises the distribution of a continuous random variable. Since ${m}_{\alpha }^{PB}(n)\rightarrow {m}_{1-\alpha }$, then

\begin{eqnarray*}\mathrm{P} \left( \mathrm{max}_{i\not = k} \left\vert \frac{{\hat {\lambda }}_{ik}-{\lambda }_{ik}}{\sqrt{\widehat{Var}({\hat {\lambda }}_{ik})}} \right\vert \leq {m}_{\alpha }^{PB}(n) \right) & \rightarrow & \mathrm{P} \left( \mathrm{max}_{i\not = k} \left\vert \frac{{W}_{i}-{W}_{k}}{\sqrt{ \frac{{\sigma }_{i}^{2}}{{\varphi }_{i}} + \frac{{\sigma }_{k}^{2}}{{\varphi }_{k}} }} \right\vert \leq {m}_{1-\alpha } \right) \end{eqnarray*}

(38) \begin{eqnarray*}& =& \mathrm{P} \left( \mathrm{max}_{i\not = k} \left\vert \frac{{W}_{i}^{\ast }-{W}_{k}^{\ast }}{\sqrt{ \frac{{\sigma }_{i}^{2}}{{\varphi }_{i}} + \frac{{\sigma }_{k}^{2}}{{\varphi }_{k}} }} \right\vert \leq {m}_{1-\alpha } \right) \nonumber\\\displaystyle & =& 1-\alpha \end{eqnarray*}

as *a* → ∞, where *m*_1−*α*_ denotes the 100(1 − *α*)^*th*^ percentile of $\text{max}_{i\not = k} \left\vert \frac{{W}_{i}^{\ast }-{W}_{k}^{\ast }}{\sqrt{ \frac{{\sigma }_{i}^{2}}{{\varphi }_{i}} + \frac{{\sigma }_{k}^{2}}{{\varphi }_{k}} }} \right\vert $. This implied that

$\text{P} \left[ {\lambda }_{ik}\in \left( {\hat {\lambda }}_{ik}\mp {M}_{\alpha }^{\text{PB}}\sqrt{\widehat{Var}({\hat {\lambda }}_{ik})} \right) \right] \rightarrow 1-\alpha $.

## Additional Information and Declarations

### Data Availability

The following information was supplied regarding data availability: The raw data and R code are available in the Supplementary File.
